# The development of an Android platform to undertake a discrete choice experiment in a low resource setting

**DOI:** 10.1186/s13690-019-0346-0

**Published:** 2019-04-17

**Authors:** Marwa Abdel-All, Blake Angell, Stephen Jan, D. Praveen, Rohina Joshi

**Affiliations:** 1 The George Institute for Global Health, Missenden Road, PO Box M 201, Camperdown, NSW 2050 Australia; 2 Sydney Medical School, University of Sydney, Sydney, New South Wales Australia; 3 Faculty of Medicine, University of New South Wales, Sydney, New South Wales Australia; 4 The George Institute for Global Health, Hyderabad, Telangana India

**Keywords:** Discrete choice experiment (DCE), Community health workers (CHW), Low-income, Cognitive testing, Android platform

## Abstract

**Background:**

Discrete choice experiment (DCE) is a quantitative technique which helps determine preferences from a definite set of choices. DCEs have been widely used to inform health services in high-income country settings and is gradually being used in low and middle-income countries (LMICs). There are challenges in deploying this method in LMIC settings due to the contextual, cultural and language related barriers. Most DCEs are conducted using paper-based tools. With mobile technology readily accessible across LMICs, we developed an Android-based platform to conduct a DCE among community health workers (CHWs) in rural India.

**Methods:**

This paper describes the development of a DCE for low-literacy community health workers (CHWs) in low-resourced setting on an Android platform. We illustrate the process of identifying realistic and locally relevant attributes, finalising the tool and cognitively testing it among respondents with an average of 10 years of education using ‘think aloud’ and ‘verbal probing’ techniques. The Android application was tested in two rounds, first by the research team and second, by the CHWs. The ‘think aloud’ and ‘verbal probing’ techniques were essential in assessing the comprehension of the CHWs to the DCE choices.

**Results:**

The CHWs did not take much time to familiarize themselves with the Android application. Compared to the paper based DCE, the time required for data collection using the Android application was reduced by 50%. We found the Android-based app to be more efficient and time saving as it reduced errors in data collection, eliminated the process of data entry and presented the data for analysis in real time.

**Conclusion:**

Electronic administration of DCE on Android computer tablets to CHWs with basic education is more efficient, time-saving than paper-based survey designs once the application is provided. It is feasible to use technology to develop and implement DCEs among participants with basic education in resource poor settings.

## Background

Discrete choice experiment (DCE) is a quantitative research method used to assess the preferences and priorities of individuals. DCEs analyse the trade-offs individuals make when choosing between sets of hypothetical choices with different attributes. It is regarded as a more useful tool than ranking techniques in policy analysis and planning as respondents are forced to explicitly make trade-offs between the attributes of differing policy alternatives [1]. DCEs have been extensively used in fields such as marketing research and transport economics. More recently, DCEs are being used in health economics to determine consumer choices. For instance, DCEs are used to understand patient or provider preferences to guide decision-making in providing customized care and to navigate policy decisions on different implementation strategies. DCEs engage a variety of stakeholders including patients, healthcare providers and administrators to identify their personal preferences. This is critical for successful design and implementation of interventions; and to minimise the gap between policy and evidence-based practices [2].

The World Health Organisation and The World Bank have developed DCE guidelines to encourage policy makers in resource poor settings in order to identify health workforce retention strategies [3]. Furthermore, DCEs are also used to inform policies to reduce health disparities in resource poor settings [4]. Research indicates that DCEs may be cognitively challenging for individuals with low literacy levels, and have recommended pre-testing the tool during the development stage to ensure that the DCE captures the true preferences of respondents [5].

Electronic data collection has been proposed as a means to present the data in a user friendly way to make the choice sets less confusing for the respondents with low literacy levels [6, 7]. The median response time for electronic data collection is shorter compared to paper-based data collection and more cost-effective, apart from the initial cost of application development [8]. However, there is limited information on the role that electronic data collection processes can play in carrying out DCEs among participants with low educational level.

In India, a cadre of CHWs, the Accredited Social Health Activist (ASHA) are remunerated according to a performance-based incentive system. Similar to other CHW based programs, inadequate remuneration and the complex nature of tasks leads to high attrition rates of the CHWs [9]. We sought to understand the set of attributes and choices acceptable to CHWs, which would promote their retention and motivate them to stay in the health systems. In this study, we describe the development of an Android platform for the administration of a DCE among CHWs in rural India.

### Methodological developments

We developed an Android-based application to display a DCE in order to quantify the relative importance of different factors, which improve job-satisfaction and motivate ASHAs in a state in rural India. The DCE development process is described below:

- Step 1: identification of attributes and levels

Literature and policy documents review along with qualitative research were essential to understand the contextual factors and challenges that the ASHAs face in delivering healthcare; and to guide the development of a list of key attributes relating to the ASHA’s job conditions. Expert consultation allowed the editing of the terms used for different levels to be more comprehensible and relevant to the ASHAs. The software “Ngene” (version 1.2.0, Choice Metrics Pty Ltd., Sydney, NSW, Australia) [10] was used to generate the DCE experimental design based on the final sets of attributes. All the documents and tools were translated into the local language (Telugu).

- Step 2: cognitive testing

A team of three field staff who were independent of the local health system conducted the cognitive testing. The team was trained on the study protocol, ethics and administration of DCE. All the surveys were administered in the local primary health centre. All the invited ASHAs agreed to participate in the DCE and provided written informed consent. The pre-test was used to assess the understanding of the DCE choice sets and concepts by the ASHAs. The cognitive testing involved administrating the DCE in a paper-based form to 20 ASHAs. We used ‘think aloud’ and ‘verbal probing’ techniques to assess the ASHA’s comprehension (ability to understand the question as intended), retrieval of information (thinking about the question and drawing conclusions), judgement and selection of response to the question. The first 10 surveys were conducted on the first day as one-to-one survey administration. The second day, a different approach was used where a group of ASHAs were seated in a classroom setting and each responded to the DCE on her own. At the beginning of the sessions, the DCE procedure and job-sets were explained after which the ASHAs completed the survey without further assistance. The team ensured that the choices were made individually and group discussion was discouraged. At the end of each data collection day, the team discussed the survey with the ASHAs to understand the strategies used to make the choices.

The field staff were asked to complete a debriefing report summarising their observations and reflections of the survey and their discussions with the ASHAs. We used this report to identify cognitive challenges faced by the ASHAs in completing the survey. Since there were no changes in the choice sets or attributes, the paper based DCE was then converted to an Android application.

- Step 3: development of the Android application

**Fig. 1:**
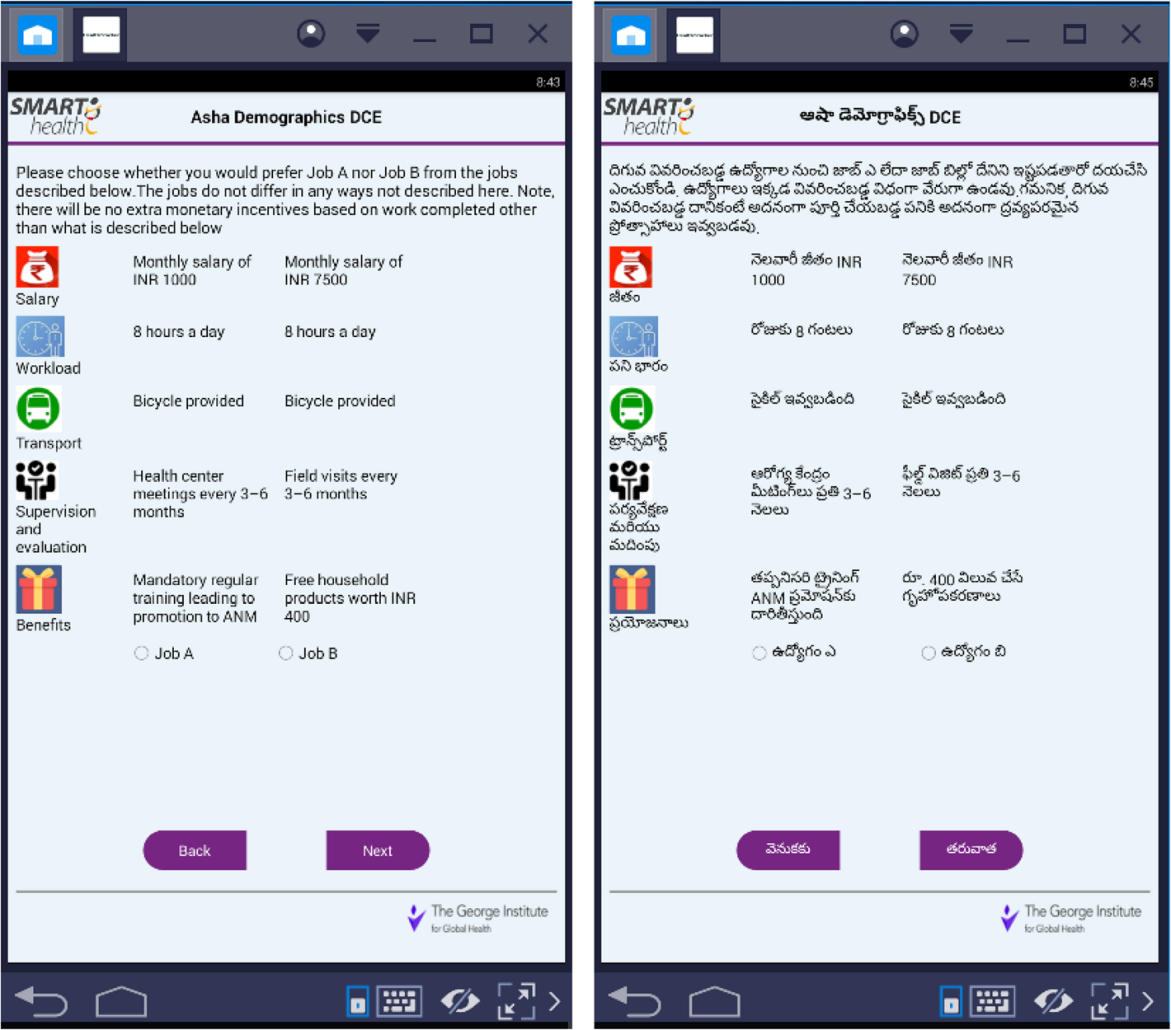
Screenshot of the English and Telugu versions of the DCE application

A detailed Microsoft Excel spreadsheet with all the demographic items and DCE choice sets was prepared. This spreadsheet imitated the expected data output required for analysis of the DCE results. All the variables were provided to the team to develop an offline Android-based application using Java programming with Android studio as an integrated development environment (IDE). Android custom layouts were used for designing the user interface and Android emulators were used as virtual device for testing the application. Data was stored locally in the internal storage of the device using SQLite database. The application was first developed in English and then into the local language (Telugu). A screenshot of both versions of the DCE is presented in Fig. 1. Samsung Tab 4 with Android version 4.2 was used to collect the information. First demographics were collected, presented in the form of dropdown choices. Next, DCE choice sets were presented with pictures and graphics to engage the respondents. The DCE choice sets required the respondents to tick their preferred job, which took them to a pop-up screen with their chosen job choice to confirm their acceptance to take the job, if offered to them. Forced response functionality, which forces the respondent to answer each question in order to proceed to the next section, was used to prevent missing data. The collected data was securely saved on the computer tablets, and later sent to the developer team to be extracted into the desired output for analysis.

### Testing of the Android application

The research team conducted the first round of user testing of the Android application to check for any errors in the sequence or display of questions. Then a second round involved user testing of the application among ASHAs to assess if they could use the mobile tablets with ease.

## Results

The average age of participated ASHAs was 31 years and 60% of them had completed secondary education. The research team conducting the survey reported that the DCE was well received by the ASHAs and that they did not find it difficult to understand the choice sets presented to them. The introductory statement with the choice set example helped to explain the hypothetical nature of the DCE and confirmed the cognitive understanding of the ASHAs of the DCE.

Data collection using the paper based format occurred over 2 days during February 2018. Once we had clarified the cognitive understanding of the choice sets, we piloted the DCE data collection using the Android application with ASHAs. This step was essential to ensure that the ASHAs could use the tablets with ease. Twenty ASHAs were invited for the pilot session, the DCE procedure and job-sets were explained to them and they were allowed to complete the DCE individually without any group discussions. The research team who observed the Android-based data collection, reported that the ASHAs did not take much time to familiarize themselves with the computer tablets. The observers noticed that the younger ASHAs used the tablets with more ease compared to the older ASHAs. Most ASHAs had never used a computer tablet prior to the study, but almost all of them had access to a smart mobile phone. The Android-based answers were in line with the paper-based tool answers. Data collection time was notably shorter using the computer tablets, (10 to 15 min) compared to (20 to 25 min) for completing the paper based DCE. In addition, data collection time was notably quicker, since no additional data entry or cleaning was required.

## Discussion

This methods paper describes the approach used to develop an Android-based DCE for CHWs with basic education in a rural region of India. The user testing of the DCE on the Android tablets showed that the ASHAs had no difficulty using the tablets especially those who had access to a smart phone. Apart from the initial cost of developing the Android application, the application required minimum user interaction with the device and allowed no skipping of questions by respondents. Data collected using the computer tablets is easily transferred to the main server to allow for real time analysis. In addition, the electronic administration of the DCE allows a better design to display the choice sets by incorporating graphics and pictures. Presenting different options using graphics is advantageous, especially when dealing with respondents with basic education.

While all studies use similar steps to develop DCEs, there are particular challenges in implementing DCEs in LMICs where the culture, context, literacy levels and language need to be considered during the design stage. Cognitive testing of the DCE is necessary to assess the respondent’s comprehension of the choice sets. In addition to literature reviews, we found qualitative research and expert consultation to be particularly helpful in identifying realistic attributes that were relevant to the local context. Mobile technology is increasingly being used in LMICs in improving public health services especially by frontline health workers [11]. Although LMICs can greatly benefit from the increasing use of the mobile phones, they can be limited by the service cost. In order to support researchers in LMICs, the United States Agency for International Development (USAID) has developed a rapid DCE tool for LMICs to simplify the design and implementation of evidence-based recruitment and retention strategies which eliminate the need of expertise [12].

It was important that the data collectors were independent of the health system, and the data collected was de-identified and confidential. This ensured that the ASHAs could trust the research team and answer the questions honestly. The two approaches used to test cognition of the questionnaire were ‘think aloud approach’ and ‘verbal probing’. The think-aloud approach is where respondents verbalize their thoughts as they try to answer the questions [13]. This helped us understand the main concerns of the ASHAs while answering the DCE. In the ‘verbal probing’ approach the interviewer asks a series of questions designed to elicit information beyond that normally provided by the respondent [14]. These two approaches helped us assess the respondent’s understanding of the options. It also explained the ASHA’s thought process and the factors used to guide their decisions regarding the jobs offered. Most of the ASHAs used smartphones in their daily routine, while using a tablet was novel to display the DCE, it was not challenging for them.

Although, one of the main advantages of using technology is connecting with remote people using network connections, this study did not investigate the capability of the low-literate community health workers to handle the complete DCE experiment without the research team guidance to explain the nature of the experiment. Our study only needed minimal interaction with the computer tablets, hence, further research need to be conducted to investigate the capabilities of community health workers to use the computer tablets on their own.

## Conclusion

This paper illustrates the steps needed to develop an Android-based application used to conduct a DCE among CHWs in rural India. The CHWs with basic education found it easy to use Android computer tablets to complete the DCEs. Data collection using an Android platform was time-saving, and more efficient compared to the paper based tools as it had inbuilt checks, eliminated data entry, and produced a data set which was clean and ready for analysis. India is one of the largest smartphone markets in the world and has extensive phone networks; this in turn can facilitate the administration of the DCEs to a wider population. In conclusion, it is feasible to use digital technology to develop and implement a DCE for CHWs with low literacy levels in a low-income setting.

## Abbreviations

ASHA: Accredited Social Health Activist
CHWs: Community Health Workers
DCE: Discrete Choice Experiment
IDE: Integrated development environment
LMIC: Low and Middle Income Countries
